# Observer-Rated Alexithymia and its Relationship with the Five-Factor-Model of Personality

**DOI:** 10.5334/pb.302

**Published:** 2016-05-26

**Authors:** Nicole Rosenberg, Michael Rufer, Vladimir Lichev, Klas Ihme, Hans-Jörgen Grabe, Harald Kugel, Anette Kersting, Thomas Suslow

**Affiliations:** 1 Department of Psychosomatic Medicine and Psychotherapy, University of Leipzig, Leipzig, Germany; 2 Department of Psychiatry and Psychotherapy, University Hospital Zurich, University of Zurich, Zurich, Switzerland; 3 Department of Psychiatry and Psychotherapy, University of Greifswald, Greifswald, Germany; 4 Department of Clinical Radiology, University of Münster, Münster, Germany

**Keywords:** alexithymia, personality, five-factor model, observer-rating, self-report

## Abstract

Studies examining the relationship between alexithymia and personality exclusively employed self-report measures of alexithymia. In the present study, we examined the relationship of both observer-rated and self-reported alexithymia with the Big Five personality dimensions. We administered the Toronto Structured Interview for Alexithymia (TSIA) as an interview-based measure of alexithymia and, in addition, two self-report questionnaires, the 20-item Toronto Alexithymia Scale (TAS-20) and the Bermond-Vorst Alexithymia Questionnaire (BVAQ). Fifty-one university students were interviewed and completed the alexithymia scales and the NEO Five-Factor Inventory. In contrast to TAS-20 and BVAQ, the Difficulty identifying feelings (DIF) scale of the TSIA was found to be unrelated to neuroticism, suggesting that the frequently reported association between DIF and neuroticism could be due to the use of self-report scales. In contrast, the affective dimension of alexithymia, measured by the BVAQ, was even negatively related with neuroticism. Thus, a paucity of fantasy and little emotional arousal goes together with increased emotional stability. Furthermore, we revealed negative correlations between interview-based alexithymia scores and openness to experience and agreeableness, which cross-validated the self-report findings. Finally, extraversion and conscientiousness each showed only one negative correlation, namely with subscales of the BVAQ. Taken together, our findings show that on the basis of interviews there is no evidence for a relation of DIF with neuroticism, while associations of alexithymia with low openness to experience and low agreeableness emerged irrespective of assessment approach. The relations of alexithymia with personality are discussed in the light of different measurement approaches.

## Introduction

Alexithymia is a personality construct characterized by difficulty identifying feelings, distinguishing between feelings and bodily sensations of emotional arousal, and describing feelings to others (Taylor, Bagby, & Parker, 1997). Furthermore, alexithymia implies a cognitive style which is oriented towards external events rather than internal processes and comes along with an impoverished fantasy life (*la pensée opératoire*; Marty & de M’Uzan, 1963). Most authors consider alexithymia as a dimensional personality trait that is distributed normally in the general population (Franz et al., 2008).

A main problem of the alexithymia construct has always been its measurement. Taylor, Ryan, and Bagby (1985) provided the first instrument meeting psychometric criteria, the Toronto Alexithymia Scale. It comprised four factors, namely: (a) difficulty identifying and distinguishing between feelings and bodily sensations (DIF); (b) difficulty describing feelings (DDF); (c) reduced daydreaming (RD); and (d) externally-oriented thinking (EOT). However, the factor ‘reduced daydreaming’ appeared to correlate negatively with the first factor (Taylor, Ryan, & Bagby, 1985). Subsequent revision of the scale resulted in the release of the TAS-20 (Bagby, Taylor, & Parker, 1994) in which the factor ‘reduced daydreaming’ was eliminated. Nevertheless, it is currently the most frequently used measure for assessing alexithymia and demonstrates good internal reliability and factorial validity (Parker, Taylor, & Bagby, 2003).

According to Vorst and Bermond (2001), alexithymia comprises a cognitive as well as an emotional component, that is, thoughts about feelings and experience of emotions, respectively, suggesting the existence of two corresponding dimensions of alexithymia. They further state, that the TAS-20 only assesses the cognitive component, and therefore developed an alternative self-report measure of alexithymia, the Bermond-Vorst Alexithymia Questionnaire (BVAQ; Vorst & Bermond, 2001). The BVAQ measures both dimensions by two higher order factors, ‘cognitive’ and ‘affective’. The affective component assesses (reduced) experience of emotional feelings (subscale ‘emotionalizing’) as well as (reduced) ability to fantasize (subscale ‘fantasizing’). Thus, it includes the fantasy facet of the alexithymia construct. Both scales are negatively oriented, that is, they assess the degree of impairment in emotionalizing and fantasizing, respectively. Several studies demonstrated the validity and reliability of the BVAQ (Müller, Bühner, & Ellgring, 2004; Vorst & Bermond, 2001).

Despite its prevalent use, the TAS-20 received some criticism. There is evidence that the scale assesses depressive and anxious tendencies as it is correlated with several measures of negative emotions (Lumley, 2000). Therefore, controlling for negative affectivity is recommended (Honkalampi et al., 2000). Most importantly, there has been doubt about the validity of self-report instruments for assessing alexithymia, as such instruments require the ability to assess one’s own difficulties in identifying and describing emotional states accurately (Lundh et al., 2002; Suslow et al., 2001). At least individuals with high degrees of alexithymia should not be able to make valid evaluations about those deficits by themselves. Many authors therefore recommend the use of alternative measures, such as interviews, at best the combination of different measurement methods (Lumley et al., 2005; Taylor & Bagby, 2004).

The Toronto Structured Interview for Alexithymia (TSIA; Bagby et al., 2006) is an observer-rated measure for assessing alexithymia. The authors provide a set of scoring criteria, prompts, and probes for each interview question, and thereby enable the interviewer to get comprehensive information from the respondent, which in turn leads to a more valid evaluation of the respondent’s answer. This is even underpinned by the requirement to give examples, which are used to verify the initial response. Importantly, the TSIA includes questions regarding impoverished fantasy in alexithymia (‘imaginal processes’, IMP, referring to reduced imagination). With regard to psychometric properties, the TSIA has shown adequate reliability and validity scores in several studies (Bagby et al., 2006; Grabe et al., 2009). The results of a recent study provided support for measurement equivalence of the English, Dutch, German, and Italian language versions of TSIA (Keefer et al., 2015).

More than four decades of research in the field of personality led to the generation of five broad trait constructs: neuroticism, extraversion, openness, agreeableness, and conscientiousness. These so-called ‘Big Five’ constitute the five-factor model of personality (FFM; e.g., Digman, 1990). Existing studies examining the relationship between alexithymia and personality exclusively employed self-report measures of alexithymia. Thus, to date research has only used self-report data and the cross-validation of these findings with other measures is lacking.

Despite the differences in personality measures and sample characteristics, there is some convergence appearing across previous studies. A major finding is the strong association between TAS-20, and especially its subscale DIF, and neuroticism. Zimmermann and colleagues (2005) reported a correlation of *r* = .52 for TAS-DIF with neuroticism in a sample of healthy students. The strong correlations found in many studies examining the relationship with personality (e.g., Bagby, Taylor, & Parker, 1994; Picardi, Toni, & Caroppo, 2005; however, see Luminet et al., 1999, for moderate correlations) indicate that high alexithymic individuals seem to be more anxious, nervous, vulnerable, and worrying than individuals low in alexithymia. As all these results have been found with one and the same alexithymia measure, namely the TAS-20, they allow for the conclusion that this could represent a method specific effect. In fact, there is evidence that the TAS-20 is a measure of general distress instead of (deficient) abilities to identify and verbalize emotions (Leising, Grande, & Faber, 2009). Besides this, Lundh et al. (2002) have argued that the TAS-20 is considerably related to perfectionism and perceived self-efficacy, independent of negative affectivity. According to the authors, high personal standards might affect the response to questions regarding one’s difficulties, that is, the degree of a perceived lack of (meta-emotional) self-efficacy influences (and artificially elevates) scores on the TAS-20. Regarding neuroticism, the strong relation with TAS-DIF could also be due to the fact that people with high personal standards and a perceived insufficiency tend to score high on neuroticism – as well as on TAS-DIF. In contrast, observer-rated measures of alexithymia should be unaffected by perfectionism, as the interviewer is able to reveal such forms of biases. Consequently, interview-derived ‘difficulty identifying feelings’ scores should not be related to neuroticism.

Consistent with the theoretical conception of the ‘pensée opératoire’ as a concrete, utilitarian thinking style (Marty & de M’Uzan, 1963), most studies reported moderate to strong negative relationships of TAS-EOT with openness to experience (Bagby, Taylor, & Parker, 1994; Wise, Mann, & Shay, 1992). Individuals high in openness are assumed to be curious, free-thinking, imaginative, and to have a permeable cognitive structure (McCrae & Costa, 1997), whereas low openness is denoted by a limited fantasy, a core characteristic of alexithymia.

Extraversion showed rather consistently negative relationships with alexithymia, but varying in size. Most studies have reported moderate to strong correlations (Luminet et al., 1999; Parker, Bagby, & Taylor, 1989; Wise, Mann, & Shay, 1992), others rather small ones (Zimmermann et al., 2005). However, in a recent study with high alexithymic individuals, Alkan Härtwig et al. (2014) did not reveal a relationship of TAS-20 with extraversion. Overall, these results suggest that alexithymia may be related to quiet, reserved behavior, and a reduced capability to experience positive emotions.

With regard to the personality traits agreeableness and conscientiousness, findings are quite inconsistent. In a sample of university students, Luminet and colleagues (1999) found no correlations of both traits with alexithymia measured by TAS-20. In contrast, Picardi, Toni, & Caroppo (2005) reported moderate negative correlations of TAS-EOT with agreeableness and conscientiousness, respectively. According to the latter study, alexithymic individuals are inclined to be more uncooperative, argumentative, and less empathic (low agreeableness), as well as impulsive, unreliable, and alienating (low conscientiousness).

Müller, Bühner, & Ellgring (2004), examining a clinical sample, and Alkan Härtwig et al. (2014), investigating a healthy sample, both applied additionally the self-report measure BVAQ which includes the fantasy and emotionalizing facet. Interestingly, both studies revealed only small to moderate positive correlations of the cognitive dimension with neuroticism. In contrast, the ‘fantasizing’ and ‘emotionalizing’ subscales, composing the affective dimension of the BVAQ, both were negatively correlated with this trait. Furthermore, Alkan Härtwig et al. found a significant positive correlation of ‘fantasizing’ and ‘analyzing’ with conscientiousness. According to these results, the BVAQ seems to reveal more facets of relations with personality than TAS-20 does.

Overall, research on alexithymia and its relationship with the FFM provides accumulating evidence for a medium to strong positive association of the cognitive aspect of alexithymia with neuroticism, that is, when alexithymia is assessed with the TAS-20 and the cognitive dimension of the BVAQ, respectively. The affective dimension rather shows a negative correlation with neuroticism. Further, there is evidence for a moderate to strong negative association of alexithymia with openness to experience. Extraversion seems to be negatively related with alexithymia, although some authors did not find such a relation. Regarding the personality traits agreeableness and conscientiousness, results are rather inconsistent. This may be explained, at least in part, by the exclusive use of self-report measures for the assessment of alexithymia, which have been criticized repeatedly.

The present study is the first that examined the relationship between observer-rated alexithymia and the five-factor model of personality. We combined self-report questionnaires (TAS-20, BVAQ) and an observer-rated measure (TSIA) to assess alexithymia. By using the BVAQ and the TSIA, relations between the fantasy facet of alexithymia and personality traits could be examined. As there is only a moderate correlation of TAS-20 and TSIA in healthy subjects (Bagby et al., 2006), it is reasonable to examine the relationship of observer-rated alexithymia with personality because there might be differences in contrast to self-reported alexithymia. Moreover, we controlled for negative affectivity (i.e., depressive symptoms and trait anxiety) as recommended in the literature (Honkalampi et al., 2000).

Based on previous findings, we hypothesized that ‘difficulty identifying feelings’ assessed by self-report correlates strongly and positively with neuroticism. In contrast, there should be no such relation for observer-rated alexithymia (TSIA-DIF). Based on findings with self-report measures, we expected the IMP scale of TSIA as well as ‘fantasizing’ to correlate moderately and negatively with neuroticism. Both scales were expected to be strongly and negatively related with openness to experience, whereas TAS-EOT should show moderate and negative correlations with openness. Furthermore, we hypothesized medium size negative correlations for extraversion, independent of alexithymia measure. Agreeableness was expected to show small negative correlations with observer-rated as well as self-report alexithymia. Finally, BVAQ scales ‘fantasizing’ and ‘analyzing’ were assumed to correlate small to moderately and positively with conscientiousness, the same holds for TSIA-IMP. In contrast, TAS-EOT and TSIA-EOT were expected to show small and negative correlations with conscientiousness.

## Method

### Participants

The sample consisted of 51 (25 women; mean age = 23.2 years, *SD* = 2.9) university students. None of them had any history of psychiatric disorders, as assessed by the Structured Clinical Interview for DSM-IV (Wittchen, Zaudig, & Fydrich, 1997). All participants gave written informed consent to participate and received financial compensation. The procedure of the study was approved by the local ethics committee and was in accordance with the declaration of Helsinki.

### Measures

#### Twenty-item Toronto Alexithymia Scale

The 20-item Toronto Alexithymia Scale (TAS-20; Bagby, Taylor, & Parker, 1994; German version: Bach et al., 1996) is a self-report measure of alexithymia. Each item is rated on a 5-point Likert scale. Total scores range from 20 to 100, with higher scores indicating higher alexithymia. It comprises three factors, namely difficulty identifying feelings (DIF), difficulty describing feelings (DDF), and externally-oriented thinking (EOT).

#### Bermond-Vorst Alexithymia Questionnaire

As a second self-report measure of alexithymia the Bermond-Vorst Alexithymia Questionnaire was used (BVAQ; Vorst & Bermond, 2001; German version: Müller, Bühner, & Ellgring, 2004), form AB, consisting of 40 items. Ratings are made on a 5-point Likert scale; the total scores range from 40 to 200, with higher scores indicating higher alexithymia. The BVAQ includes five factors: ‘emotionalizing’, ‘fantasizing’, ‘identifying’, ‘analyzing’, and ‘verbalizing’. ‘Emotionalizing’ and ‘fantasizing’ compose the *affective* dimension of alexithymia; ‘identifying’, ‘analyzing’ and ‘verbalizing’ the *cognitive* dimension (Bermond et al., 2010).

#### Toronto Structured Interview for Alexithymia

As an observer-rated measure for alexithymia we used the Toronto Structured Interview for Alexithymia (TSIA; Bagby et al., 2006; German version: Grabe et al., 2014). It consists of 24 questions relating to the factors difficulty identifying feelings (DIF), difficulty describing feelings (DDF), externally oriented thinking (EOT), and imaginal processes (IMP). The first two factors compose the domain scale affect awareness (AA), the second two factors the domain scale operative thinking (OT). The answers of the respondents are rated by a trained interviewer on a 3 point scale; total scores ranging from 0 to 48 with higher scores indicating higher alexithymia.

#### NEO Five-Factor Inventory

Personality was assessed with the NEO Five-Factor Inventory (NEO-FFI; Costa & McCrae, 1992; German version: Borkenau & Ostendorf, 2008). This widely used self-report questionnaire consists of 60 items measuring five domains of personality: neuroticism, extraversion, openness to experience, agreeableness, and conscientiousness. Scoring is made on a 5-point Likert scale with higher scores indicating a higher level of that personality dimension. The NEO-FFI shows satisfactory to good reliability (Cronbach’s alpha between .72 and .87) and high stability (test-retest across five years between .71 and .82). The validity of the test has been demonstrated in several studies (Borkenau & Ostendorf, 2008).

#### Beck Depression Inventory

To control for depressive symptoms the Beck Depression Inventory II was administered (BDI-II; Beck, Steer, & Brown, 1996; German version: Hautzinger, Keller, & Kühner, 2006), which is a 21-item questionnaire. Total scores range from 0 to 63 with higher scores indicating more severe depressive symptoms. The BDI-II is a frequently used measure with good psychometric properties in clinical and nonclinical samples (Cronbach’s alpha > .88; Hautzinger, Keller, & Kühner, 2006).

#### State-Trait-Anxiety Inventory

Trait anxiety was assessed using the trait version of the State-Trait-Anxiety Inventory (STAI-T; Spielberger, Gorsuch, & Lushene, 1970; German version: Laux et al., 1981). It contains 20 items with total scores ranging from 20 to 80; higher scores indicating stronger trait anxiety. The STAI-T shows high internal consistency (Cronbach’s alpha > .88) and has proven both construct and criterion validity (Laux et al., 1981).

### Procedure

Participants were informed about the study and asked for giving written informed consent. Initially, the Structured Clinical Interview for DSM-IV (SKID I; German version: Wittchen, Zaudig, & Fydrich, 1997) was conducted for detection of past or present psychiatric disorders. When free of mental disorders, subjects were included in the study and completed the TAS-20 and the BVAQ. Afterwards, the TSIA interview was conducted by one interviewer who also did the ratings. To improve quality of assessments by inter-rater reliability, all interviews were video recorded and subsequently rated by a second person. Both raters were familiar with the alexithymia construct. To become acquainted with the TSIA, they read a manual outlining administration and scoring procedures for the TSIA (Grabe et al., 2014). The interviewer and the second rater were trained in the administration and scoring of the interview by one of the translators of the German version of the TSIA, including discussion of the scoring guidelines and the correct use of prompts and probes. Additionally, the quality of interview administration was checked by one of the translators of the German version who gave feedback about a set of test-interviews. To assess level of agreement, we used weighted kappa, because level of measurement was ordinal and only two raters were involved. Estimates exceeding 0.60 are considered as adequate inter-rater reliability (Altman, 1991). Due to technical problems, one interview was not video-recorded, thus, inter-rater reliability could only be calculated for *N* = 50 interviews. The estimated weighted kappa for the TSIA total score was *k* = .67, which suggests an adequate level of agreement. At the time of rating, both raters were not informed about TAS-20 and BVAQ scores of the participants. Interviews were done on an individual basis and lasted about 90 minutes. Later on, NEO-FFI, BDI-II and STAI-T were filled out.

### Statistical analyses

Means and standard deviations for the scales of TSIA, BVAQ, TAS-20, and NEO-FFI were calculated. Pearson product-moment correlations between TSIA, BVAQ, and TAS-20 were determined to examine the pattern of relationships among the different measures of alexithymia. Relationships between alexithymia measures and personality traits were assessed by calculating Pearson product-moment correlations between scales and domains of TSIA, BVAQ, TAS-20, and NEO-FFI. To take into account possible influences of depressive symptoms and trait anxiety on alexithymia, we also calculated partial correlations with BDI-II and STAI-T scores as control variables. This did not change the results substantially, so we do not present the results here. All analyses were conducted using IBM SPSS Statistics 20.0. To compare the sizes of the different correlations we calculated Steiger’s *Z* statistics (Steiger, 1980) for the corresponding alexithymia subscales (i.e., TSIA-DIF compared with ‘identifying’ and TAS-DIF, TSIA-DDF with ‘verbalizing’ and TAS-DDF, TSIA-EOT with ‘analyzing’ and TAS-EOT, TSIA-IMP with ‘fantasizing’) by using formulas implemented in Lee and Preacher (2013). Differences at *p* < .05 (two-tailed; one-tailed in case of specific (directed) hypotheses) were considered as statistically significant.

## Results

### Descriptive statistics

Mean scores, standard deviations, and ranges of TSIA, BVAQ, TAS-20, and NEO-FFI scales are presented in Table 1.

**Table 1:** Descriptive statistics: means, standard deviations, and ranges for the alexithymia measures and the personality measure.

Scale	Mean	SD	Range

TSIA Total	16.7	9.7	2–37
TSIA-AA	4.2	5.2	0–19
TSIA-OT	12.6	5.6	2–23
TSIA-DIF	1.4	2.1	0–8
TSIA-DDF	2.8	3.3	0–11
TSIA-EOT	5.8	3.3	0–12
TSIA-IMP	6.8	2.9	1–11
BVAQ Total	106.3	23.9	65–166
BVAQ-AFF	45.8	10.3	28–71
BVAQ-COG	60.5	17.2	28–99
Verbalizing	23.0	7.9	9–39
Fantasizing	25.7	7.2	13–37
Identifying	18.8	5.8	8–36
Emotionalizing	20.1	6.2	11–35
Analyzing	18.8	6.5	9–32
TAS-20 Total	43.2	10.9	22–71
TAS-DIF	12.6	4.5	7–25
TAS-DDF	12.4	4.6	5–24
TAS-EOT	18.2	4.6	10–31
Neuroticism	14.8	6.7	3–29
Extraversion	29.8	7.3	13–44
Openness	29.5	6.5	15–42
Agreeableness	34.0	5.8	19–45
Conscientiousness	34.1	6.8	13–47

N = 51. TSIA: Toronto Structured Interview for Alexithymia; AA: affect awareness; OT: operative thinking; DIF: difficulty identifying feelings; DDF: difficulty describing feelings; EOT: externally oriented thinking; IMP: imaginal processes; BVAQ: Bermond-Vorst Alexithymia Questionnaire; AFF: affective composite = fantasizing + emotionalizing; COG: cognitive composite = verbalizing + analyzing + identifying; TAS-20: 20-item Toronto Alexithymia Scale.

Scores for TSIA and BVAQ are comparable to those of previous studies with clinical and non-clinical samples (Bermond et al., 2007; Bagby et al., 2006). For the TAS-20, scores are also comparable to those of other studies with non-clinical student samples (Zimmermann et al., 2005). Regarding personality traits, scores for NEO-FFI are similar to norms of a German sample of young adults (Körner et al., 2008), except for neuroticism scores, which are rather low in our study. This might be due to the fact that we screened our sample extensively for depressive tendencies as well as past and present psychiatric conditions.

### Correlational analyses

#### Correlations between alexithymia measures

Correlations between alexithymia measures are displayed in Table 2. In the following we will only address some of the significant correlations in the text. There are significant positive correlations of medium to large effect size between almost all scales of the alexithymia measures. However, the fantasy facet of alexithymia shows a different pattern of correlations: TSIA-IMP manifests no significant correlation with any of the other subscales except for ‘fantasizing’, which is not surprising as both scales assess the same facet. Besides this, ‘fantasizing’ is significantly positively correlated with the EOT-subscale (both TAS and TSIA) as well as with TAS-DDF.

**Table 2:** Correlations between measures of alexithymia.

Scale	1	2	3	4	5	6	7	8	9	10	11	12	13	14	15	16	17	18	19

1. TSIA Total		.89**	.91**	.79**	.89**	.92**	.70**	.60**	.58**	.48**	.48**	.40**	.23	.51**	.48**	.44**	.18	.39**	.50**
2. TSIA-AA			.63**	.93**	.97**	.74**	.36*	.61**	.47**	.57**	.52**	.19	.41**	.54**	.50**	.53**	.37**	.46**	.44**
3. TSIA-OT				.52**	.65**	.91**	.88**	.47**	.58**	.31*	.35*	.51**	.02	.37**	.38**	.28*	–.03	.25	.45**
4. TSIA-DIF					.81**	.64**	.28*	.46**	.31*	.45**	.37**	.09	.40**	.41**	.39**	.41**	.37**	.32*	.30*
5. TSIA-DDF						.75**	.38**	.65**	.53**	.59**	.58**	.25	.38**	.59**	.53**	.56**	.34*	.51**	.50**
6. TSIA-EOT							.61**	.54**	.53**	.44**	.46**	.33*	.14	.50**	.47**	.40**	.10	.34*	.53**
7. TSIA-IMP								.29*	.51**	.10	.15	.60**	–.12	.15	.19	.07	–.17	.09	.26
8. BVAQ Total									.78**	.93**	.86**	.49**	.59**	.72**	.87**	.83**	.48**	.81**	.69**
9. BVAQ-AFF										.48**	.46**	.80**	.14	.72**	.58**	.46**	.11	.47**	.53**
10. BVAQ-COG											.92**	.19	.74**	.57**	.86**	.87**	.61**	.85**	.64**
11. Verbalizing												.24	.52**	.48**	.74**	.83**	.51**	.89**	.59**
12. Fantasizing													–.08	.17	.29*	.22	–.09	.29*	.31*
13. Identifying														.32*	.43**	.64**	.71**	.49**	.36*
14. Emotionalizing															.63**	.51**	.28*	.44**	.51**
15. Analyzing																.72**	.35*	.71**	.66**
16. TAS Total																	.78**	.84**	.78**
17. TAS-DIF																		.52**	.37**
18. TAS-DDF																			.51**
19. TAS-EOT																			

N = 51. TSIA: Toronto Structured Interview for Alexithymia; AA: affect awareness; OT: operative thinking; DIF: difficulty identifying feelings; DDF: difficulty describing feelings; EOT: externally oriented thinking; IMP: imaginal processes; BVAQ: Bermond-Vorst Alexithymia Questionnaire; AFF: affective composite = fantasizing + emotionalizing; COG: cognitive composite = verbalizing + analyzing + identifying; TAS-20: 20-item Toronto Alexithymia Scale. *p < .05, **p < .01.

#### Correlations between personality traits and alexithymia measures

Correlations between personality traits, as measured by NEO-FFI, and different alexithymia measures are shown in Table 3.

**Table 3:** Correlations between NEO-FFI personality dimensions and measures of alexithymia.

	Neuroticism	Extraversion	Openness	Agreeableness	Conscientiousness

TSIA Total	–.09	–.15	–.39**	–.30*	–.01
TSIA-AA	.06	–.21	–.19	–.27	–.04
TSIA-OT	–.21	–.06	–.50**	–.27	.03
TSIA-DIF	.07	–.23	–.13	–.16	–.03
TSIA-DDF	.05	–.19	–.22	–.32*	–.05
TSIA-EOT	–.16	–.10	–.38**	–.31*	–.09
TSIA-IMP	–.21	.00	–.53**	–.16	.16
BVAQ total	–.01	–.14	–.46**	–.56**	–.08
BVAQ-AFF	–.30*	.05	–.41**	–.45**	.07
BVAQ-COG	.16	–.23	–.39**	–.51**	–.15
Verbalizing	.16	–.28*	–.38**	–.51**	–.08
Fantasizing	–.25	.12	–.48**	–.21	.20
Identifying	.29*	–.11	–.17	–.26	–.35*
Emotionalizing	–.21	–.05	–.12	–.49**	–.11
Analyzing	–.03	–.17	–.40**	–.48**	.01
TAS-20 total	.16	–.11	–.26	–.38**	–.08
TAS-DIF	.39**	–.09	.01	–.05	–.10
TAS-DDF	.05	–.11	–.33*	–.51**	.02
TAS-EOT	–.05	–.07	–.30*	–.35*	–.10

N = 51. TSIA: Toronto Structured Interview for Alexithymia; AA: affect awareness; OT: operative thinking; DIF: difficulty identifying feelings; DDF: difficulty describing feelings; EOT: externally oriented thinking; IMP: imaginal processes; BVAQ: Bermond-Vorst Alexithymia Questionnaire; AFF: affective dimension = fantasizing + emotionalizing; COG: cognitive dimension = verbalizing + analyzing + identifying; TAS-20: 20-item Toronto Alexithymia Scale. *p < .05, **p < .01, two-tailed.

Regarding the TSIA interview, TSIA-DIF and TSIA-DDF did not correlate with neuroticism. Furthermore, TSIA-IMP showed a negative correlation with neuroticism, that is, impoverished fantasy goes along with less neuroticism. Although non-significant, this correlation was of moderate size. There were small to moderate negative correlations of TSIA-DIF and TSIA-DDF with extraversion and openness, respectively, but again, these correlations failed to reach significance. The domain ‘operative thinking’ (TSIA-OT, including TSIA-EOT and TSIA-IMP) exhibited a strong negative relation with openness. For agreeableness, we found significant negative correlations with TSIA-DDF and TSIA-EOT, while for conscientiousness, there were no significant correlations with TSIA.

The subscale ‘identifying’ of BVAQ was found to be significantly and positively correlated with neuroticism. Similar to TSIA-IMP, there was a moderate negative, but non-significant correlation of ‘fantasizing’ with neuroticism, meaning that reduced ability to fantasize was related to low scores on neuroticism. However, the negative correlation of the affective dimension of alexithymia (BVAQ-AFF, including ‘fantasizing’ and ‘emotionalizing’) with neuroticism became significant (*r* = -.30, *p* < .05). For extraversion, a significant negative correlation was observed with ‘verbalizing’. Considering openness, there were significant negative correlations with ‘verbalizing’, ‘fantasizing’, and ‘analyzing’. Furthermore, the subscales ‘verbalizing’, ‘emotionalizing’, and ‘analyzing’ showed significant negative correlations of strong size with agreeableness. Finally, ‘identifying’ was the only alexithymia scale that was significantly and negatively related to conscientiousness.

Regarding TAS-20, TAS-DIF showed the highest (positive) correlation with neuroticism (*r* = .39, *p* < .01). There were no significant correlations of TAS with extraversion and conscientiousness, respectively. Regarding openness, we found moderate significant and negative correlations with TAS-DDF and TAS-EOT. Furthermore, both scales showed also significant negative correlations with agreeableness.

To compare sizes of correlations, we calculated Steiger’s *Z* for the corresponding alexithymia subscales. Specifically, we compared TSIA-DIF with ‘identifying’ and TAS-DIF, TSIA-DDF with ‘verbalizing’ and TAS-DDF, TSIA-EOT with ‘analyzing’ and TAS-EOT, and TSIA-IMP with ‘fantasizing’. For the sake of readability, we report in the following only significant results. Regarding neuroticism, correlation coefficients of TSIA-DIF and TAS-DIF differed significantly (*Z* = 2.09, *p* < .05), that is, TSIA-DIF was significantly less correlated with neuroticism compared to TAS-DIF. ‘Verbalizing’ was significantly stronger correlated with extraversion than TAS-DDF was (*Z* = –2.58, *p* < .01). Correlation of ‘identifying’ with agreeableness was stronger than that of TAS-DIF with agreeableness, although this difference was only marginally significant (*Z* = –1.96, *p* = .05). Furthermore, ‘identifying’ was significantly stronger correlated with conscientiousness than TSIA-DIF and TAS-DIF with this trait, respectively (*Z* = –2.11, *p* < .05 and *Z* = –2.38, *p* < .05).

## Discussion

The present study is the first to investigate the relationship between observer-rated alexithymia and the five-factor model of personality. We applied the TSIA to assess alexithymic tendencies along with two self-report measures, TAS-20 and BVAQ. In this way, it could be investigated whether relationships between facets of the alexithymia construct and the Big Five personality dimensions exist independently from assessment approach.

Correlations between the different alexithymia measures were quite high, which means that, in general, all measures are closely related to each other and tap into the same construct. However, there is one exception, namely the (impoverished) fantasy facet of alexithymia, that is, TSIA-IMP and ‘fantasizing’. We found no relation of this facet with self-reported DIF and only one significant correlation with self-reported DDF. Inter-correlations among the scales assessed with the observer-rated measures (i.e., TSIA-IMP with TSIA-DIF and TSIA-DDF) are somewhat higher, owing to the fact that measures assessed with the same method tend to correlate stronger with one another than with measures for which a different method has been used. Thus, it seems that the fantasy facet of alexithymia is somewhat independent from the other facets. Importantly, we found a strong positive correlation between observer-rated fantasy proneness, TSIA-IMP, and the corresponding self-report scale, BVAQ-‘fantasizing’ (*r* = .60), suggesting that both scales assess the same construct.

Confirming our hypothesis, ‘difficulty identifying feelings’ assessed by the TSIA was found to be unrelated to neuroticism. Previous studies based on self-report measures have reported strong positive correlations of alexithymia with neuroticism (e.g., Bagby, Taylor, & Parker, 1994). In our study, we also observed a positive correlation of TAS-DIF and BVAQ-identifying with neuroticism. In other words, when alexithymia is rated by an interviewer, no association of alexithymia with neurotic traits like anxious, vulnerable, and depressive tendencies is revealed. In contrast, when alexithymia is assessed by self-report, there is a positive relationship of alexithymia with neuroticism. The present results indicate that the correlation of alexithymia with neuroticism may represent a method-specific effect, and that both personality constructs are indeed not that close as thought so far. Our findings are also in line with the assumption that the TAS-20 could be a measure of general distress (Leising, Grande, & Farber, 2009; Rief, Heuser, & Fichter, 1996). However, as we controlled for negative affectivity, our results also allow for the explanation that the relation of difficulty identifying feelings assessed by self-report with neuroticism might be due to a negative cognitive bias owing to perfectionism (see Lundh et al., 2002, for a discussion of alexithymia and perfectionism). That is, people with high personal standards could assess their own abilities to identify their feelings in a distorted way. Because of their perceived insufficiency, they might agree more often with the deficit-oriented items of self-report questionnaires, and, at the same time score high on neuroticism. This is in line with the finding that perfectionistic concern is robustly associated with neuroticism (Dunkley, Blankstein, & Berg, 2012).

In contrast to the above discussed results, the affective component (‘fantasizing’ and ‘emotionalizing’) of the BVAQ was negatively correlated with neuroticism. This result substantiates recent findings (Alkan Härtwig et al., 2014) and suggests a benefit of having little imagination and a low level of emotion-induced arousal. As fantasy-proneness (Waldo & Merritt, 2000) and emotional reactivity (Macatee & Cougle, 2013) are associated with psychopathology, the ability to be pragmatic and to keep calm in complex situations may act as a protective factor against stress and anxiety.

One can raise the question whether one should theoretically expect alexithymia, and more specifically difficulty identifying feelings, to be correlated with neuroticism after all. Neuroticism is mainly defined by the opposite of emotional stability, that is, it comprises not only the disposition to develop negative affective states such as anxiety and depression. Instead, it also includes emotional reactivity in a broader sense and comprises the facets vulnerability, impulsiveness, and self-consciousness (McCrae & Costa, 1987). Thus, one has to distinguish at least in part between negative affectivity and neuroticism. It might be, that alexithymia is related to experiencing less positive affects – but actually it should theoretically be related to experiencing less affects *at all*. Therefore, one should expect alexithymia to be negatively associated with neuroticism. This assumption is consistent with recent neuroimaging results pointing to a hypo-responsiveness to emotion cues in alexithymia (i.e., decreased brain activity in the amygdala, insula, and precuneus during emotion processing; for a review see van der Velde et al., 2013). Findings of psychophysiological studies also indicate a decreased (autonomic) response to emotion stimuli in alexithymia (Pollatos et al., 2008), providing further evidence for the hypo-responsiveness hypothesis. According to this, alexithymia is related to a diminished neural reactivity to emotional stimuli and reduced emotional arousal, which may result in a reduced experiencing of emotions making their recognition more difficult.

The subscale ‘emotionalizing’ of the BVAQ exactly points at this issue as it assesses “the degree to which someone is emotionally aroused by emotion-inducing events” (Vorst & Bermond, 2001), in other words, the (lack of) experiencing emotional feelings. Indeed, ‘emotionalizing’ was found to correlate negatively with neuroticism, that is, people who have deficiencies in developing emotional responses show low scores on neuroticism. Although this correlation failed to reach significance (*r* = –.21, *p* < .15), which was likely due to our relatively small sample, it hints at an interesting relationship and substantiates previous findings (Alkan Härtwig et al., 2014; Müller, Bühner, & Ellgring, 2004). In our view, the ‘emotionalizing’ facet is of great importance as it represents a core feature of the alexithymia construct originally defined by Nemiah and Sifneos (1970), namely the reduced ability to experience emotional feelings, which was never included in the TAS. Instead, the TAS assesses only the cognitive aspects of alexithymia, like thinking about and dealing with one’s emotions. Thus, most of the existing studies investigating alexithymia and its relationship with personality did not assess the degree to which a person actually develops emotional reactions.

Furthermore, the (impaired) ‘imaginal processes’ scale of the TSIA, as well as ‘fantasizing’, both showed a strong negative correlation with openness to experience. This is not surprising, as alexithymic subjects are often described as practical, inflexible, and rigid, which corresponds to low openness. As McCrae and Costa (1997) have pointed out, artists and poets are prime examples of individuals high in openness, because they combine keen imaginations, sensitivity, and passion, they are curious, free-thinking, and exhibit a wide range of emotional reactivity. All of these characteristics seem to be lacking in alexithymia. On these grounds, the importance of the paucity of imagination and fantasy life as one of the main characteristics of alexithymia (Nemiah, 1977) is emphasized. Yet, other alexithymia scales, namely ‘verbalizing’ and ‘analyzing’, as well as TAS-DDF and TAS-EOT showed also significant negative relationships with openness. However, these correlations were smaller than the ones discussed above, although the difference between correlations did not reach statistical significance.

Instead of the expected small correlation, we found TSIA-DDF and TSIA-EOT to be moderately and negatively correlated with agreeableness. According to this, alexithymia is related to uncooperative and critical interpersonal behavior, as well as less empathy. It is known that alexithymia is associated with impairments in empathy (Bird et al., 2010). Importantly, in our study we also found negative relations of ‘verbalizing’, ‘analyzing’, TAS-DDF, and TAS-EOT with agreeableness, as such replicating findings of previous studies (Picardi, Toni, & Caroppo, 2005; Schäfer et al., 2002). Hence, for openness and agreeableness, subjective and objective measures of alexithymia showed very similar correlation results.

Regarding the personality traits extraversion and conscientiousness, we found only two significant correlations with alexithymia, each with subscales of the BVAQ. Extraversion was negatively related with ‘verbalizing’, suggesting that subjects who have difficulties finding words for their feelings are more introverted, quiet and shy. Previous studies report similar results (Alkan Härtwig et al., 2014; Müller, Bühner, & Ellgring, 2004), that is, a negative relation of ‘verbalizing’ with extraversion. However, regarding the corresponding scale of the TAS, namely TAS-DDF, the existing literature shows different results: some authors report no significant correlations with extraversion (e.g., Alkan Härtwig et al., 2014; Schäfer et al., 2002; Zimmermann et al., 2005), others did find significant negative correlations (Bagby, Taylor, & Parker, 1994; De Gucht, Fontaine, & Fischler, 2004; Müller, Bühner, & Ellgring, 2004). In our view, the discrepancy between BVAQ and TAS-20 might be explained by the fact that difficulty finding words for one’s feelings, measured by the BVAQ, is mainly assessed through questions relating to social interaction and gregariousness (e.g., ‘I like to tell others about how I feel’ [negative]), whereas the TAS-DDF scale does rarely imply this social aspect (e.g., ‘I am able to describe my feelings easily’ [negative]). Hence, BVAQ- ‘verbalizing’ is more aimed at the aspect of communicating with others, which in turn reflects a core feature of extraversion. Difficulty describing feelings assessed by the TSIA did not reveal a relationship with extraversion as well, which is not surprising, as the interview assesses the ability to put feelings into words directly by requiring the respondent to do so.

In our study, the main finding regarding conscientiousness (similar to extraversion) is a null result. Though conscientiousness was significantly and negatively related with the ‘identifying’-subscale of the BVAQ, TAS-DIF and TSIA-DIF did not reveal a relation with this trait. Again, this finding might be explained by the phrasing of the BVAQ-‘identifying’ items, which imply the performance aspect of conscientiousness, e.g., ‘When things get on top of me, I mostly know why’ (negative). People who often feel overstrained might tend to approve these items and at the same time score low on conscientiousness, which is characterized by ambitious, determined, systematic and responsible behavior (Borkenau & Ostendorf, 2008; Hogan & Ones, 1997). Previous studies confirm this assumption in part, as Alkan Härtwig et al. (2014) and Müller, Bühner, and Ellgring (2004) both found negative relations of ‘identifying’ with conscientiousness. However, at least in their studies, the same was true for TAS-DIF. Nonetheless, there are some studies reporting no significant relation of TAS-DIF with conscientiousness (e.g., Picardi, Toni, & Caroppo, 2005; Zimmermann et al., 2005) which is in line with our findings.

Finally, one has to keep in mind that although we found these relations of BVAQ with extraversion and conscientiousness, they concern only one scale each and are in the range of a medium effect. To put it another way, a lot of scales and facets did *not* show a significant relationship with these traits, irrespective of the kind of measure, that is, observer-rated or self-report.

The results of the present study draw the following personality profile associated with alexithymia. The most robust finding is the negative relation of alexithymia with openness to experience, shown for observer-rated as well as self-report measures. According to this, alexithymia is related to practical, logical, and conservative views with diminished imagination, sensibility, and curiosity. High alexithymic individuals prefer a down-to-earth attitude, perhaps because they tend to feel uncomfortable with complexities. Moreover, alexithymia, especially difficulties describing feelings, is related to less agreeableness, which is demonstrated by a lack of empathy and little prosocial behavior. We did not find robust relations with extraversion and conscientiousness; however, alexithymic individuals might be prone to more introverted and alienating behavior. Finally, difficulties identifying feelings, one of the core characteristics of alexithymia, is related to neuroticism only when measured by self-report. In contrast, we do not find this association with observer-rated measures, that is, the TSIA. Thus, the relation between DIF and emotional instability is measure-dependent and not generally detectable. Furthermore, the negative relation of ‘emotionalizing’ and ‘fantasizing’ as well as TSIA-IMP with neuroticism suggests that a reduced ability to experience emotional feelings and an impoverished imagination go along with emotional stability. As this result was found with self-report (BVAQ) as well as with observer-rated (TSIA) measures, it seems rather robust.

Some limitations of the present study should be noted. First, our sample consisted of students and was relatively small. Future studies should investigate more heterogeneous samples with more subjects. Second, we studied a non-clinical, young, and well educated population in which alexithymia scores were not very high. Future research should try to include more subjects with high levels of alexithymia in their samples.

Taken together, our results suggest that both, BVAQ and TSIA, provide a broader assessment of the alexithymia construct than TAS-20 as they include the fantasy facet. Only the BVAQ assesses the degree to which one experiences emotional feelings (‘emotionalizing’). This facet, together with the fantasy facet (‘fantasizing’ and TSIA-IMP) reveals an important relation with neuroticism, namely a negative one, suggesting a presumably protective function of having a paucity of fantasy. Furthermore, as the ‘difficulty identifying feelings’ scale of TSIA is unrelated to neuroticism (in contrast to TAS-DIF and BVAQ-‘identifying’), it might allow assessment of problems in the recognition of one’s own feelings independent of a negative cognitive style and perfectionistic concern. Relations with openness and conscientiousness were less affected by the alexithymia measure, however, the fantasy facet showed stronger correlations with openness than any TAS-20 scale and, further, tended to reveal positive relations with conscientiousness, which no other scale did. Regarding extraversion and agreeableness, self-report and observer-rated measures yielded quite the same results.

Thus, if one is interested in measuring a broad and differential picture of personality in alexithymia, the use of TSIA and/or BVAQ can be recommended. The observer-rated measure offers the advantage of being unrelated to negative response biases.
